# The p53 Tumor Suppressor Is Stabilized by Inhibitor of Growth 1 (ING1) by Blocking Polyubiquitination

**DOI:** 10.1371/journal.pone.0021065

**Published:** 2011-06-22

**Authors:** Subhash Thalappilly, Xiaolan Feng, Svitlana Pastyryeva, Keiko Suzuki, Daniel Muruve, Daniel Larocque, Stephane Richard, Matthias Truss, Andreas von Deimling, Karl Riabowol, Gesche Tallen

**Affiliations:** 1 Departments of Biochemistry and Molecular Biology and Oncology, University of Calgary, Calgary, Alberta, Canada; 2 Department of Medicine, University of Calgary, Calgary, Alberta, Canada; 3 Preclinical Research Team on Neurodegenerative Diseases, Chronic Disorders DAP, GlaxoSmithKline Biologicals North America, Laval, Quebec, Canada; 4 Department of Medicine and Oncology, Lady Davis Institute, McGill University, Montreal, Quebec, Canada; 5 Laboratory for Molecular Biology, Department of Pediatrics, Charité - Universitätsmedizin Berlin, Berlin, Germany; 6 Department of Neuropathology, Institute of Pathology, Ruprecht-Karls University Heidelberg and Clinical Cooperation Unit Neuropathology, Heidelberg, Germany; 7 Department of Pediatric Oncology/Haematology, Charité - Universitätsmedizin Berlin, Berlin, Germany; Istituto Dermopatico dell'Immacolata, Italy

## Abstract

The INhibitor of Growth tumor suppressors (ING1-ING5) affect aging, apoptosis, DNA repair and tumorigenesis. Plant homeodomains (PHD) of ING proteins bind histones in a methylation-sensitive manner to regulate chromatin structure. ING1 and ING2 contain a polybasic region (PBR) adjacent to their PHDs that binds stress-inducible phosphatidylinositol monophosphate (PtIn-MP) signaling lipids to activate these INGs. ING1 induces apoptosis independently of p53 but other studies suggest proapoptotic interdependence of ING1 and p53 leaving their functional relationship unclear. Here we identify a novel ubiquitin-binding domain (UBD) that overlaps with the PBR of ING1 and shows similarity to previously described UBDs involved in DNA damage responses. The ING1 UBD binds ubiquitin with high affinity (K_d_∼100 nM) and ubiquitin competes with PtIn-MPs for ING1 binding. ING1 expression stabilized wild-type, but not mutant p53 in an MDM2-independent manner and knockdown of endogenous *ING1* depressed p53 levels in a transcription-independent manner. ING1 stabilized unmodified and six multimonoubiquitinated forms of wild-type p53 that were also seen upon DNA damage, but not p53 mutants lacking the six known sites of ubiquitination. We also find that ING1 physically interacts with herpesvirus-associated ubiquitin-specific protease (HAUSP), a p53 and MDM2 deubiquitinase (DUB), and knockdown of HAUSP blocks the ability of ING1 to stabilize p53. These data link lipid stress signaling to ubiquitin-mediated proteasomal degradation through the PBR/UBD of ING1 and further indicate that ING1 stabilizes p53 by inhibiting polyubiquitination of multimonoubiquitinated forms via interaction with and colocalization of the HAUSP-deubiquitinase with p53.

## Introduction

The **IN**hibitor of **G**rowth 1 (ING1) type II-tumor suppressor [1] is down-regulated in many human malignancies [2], [3]. It is one of a family of 5 genes (*ING1* to *ING5*), several of which generate multiple protein isoforms, such as p47^ING1a^ (ING1a), p33^ING1b^ (ING1b) and p24^ING1c^ (ING1c). ING1b is the major isoform expressed in human cells [2] and is involved in tumorigenesis [2]–[4], senescence [5], apoptosis [6] and DNA repair [2], [3], [7]. Hereafter, reference to ING1 will mean ING1b unless otherwise noted. INGs function through direct interaction with other proteins [8], primarily in the nucleus [9], where they regulate chromatin structure [10]. INGs bind lamin A via their unique lamin interaction domain (LID) contributing to the Hutchinson-Gilford progeria-syndrome phenotype [11]. The most highly conserved domain of the ING proteins is their plant homeodomain (PHD), a form of zinc finger. PHDs in INGs interact with core histone proteins in a histone methylation-sensitive manner, implicating ING proteins as interpreters of the histone epigenetic code [12]–[14]. This mechanism is well-conserved considering that progressive methylation of yeast histone H3K4 also increases ING histone affinity [14]–[16]. ING2 directs the acetylation of histone H3-residue K14 [16], suggesting that INGs regulate the histone code by linking histone methylation to -acetylation. Additionally, the polybasic region (PBR) adjacent to the ING2-PHD is necessary and sufficient for binding stress-inducible phosphoinositide (PI) signaling lipids that activate ING2 to promote apoptosis [17]. Of all ING proteins, ING2 shares highest sequence-homology [18] and most functional similarities with ING1 [10].

ING1 and ING2 enhance acetylation of p53 on lysine-residues that are linked to p53-activation [19] and inactivated by hSir2 [20]. Binding of ING1 to p53 was reported to be required for p53-activity [21] and may prevent binding of the MDM2 ubiquitin E3-ligase to p53, thereby preventing proteasomal degradation of p53 [22]. However, ING1 also induces apoptosis independently of p53 [23], [24]. Hence, whether significant interactions between endogenous p53 and ING1 occur *in vivo* requires clarification.

The ubiquitin-proteasome pathway regulates levels, activity and location of about 80% of growth-regulatory proteins and transcription factors with short half-lives [25], such as cyclins, p21^WAF1^ and p53, through a network of ubiquitin-transferring proteins, ubiquitin E2 and E3-ligases, and proteins regulating their activity [26]. Most commonly, proteins are polyubiquitinated, targeting them for rapid degradation by the 26S-proteasome, while monoubiquitination and multi-monoubiquitination have been implicated in cellular stress responses, in chromatin remodeling and in regulating p53-stability [27]–[30]. Alterations in ubiquitination are frequent in cancer cells [31]. Various studies on proteasome-inhibitors in cancer treatment already show promising results, but it currently remains unclear, why blocking “non-specific” proteasomal degradation induces differential killing of tumor cells [31]. However, induction of p53-dependent apoptosis is involved in the selective killing of tumor cells by certain proteasome-inhibitors [32]. Therefore, identifying mechanisms that shield p53 from proteasomal degradation might contribute to optimized cancer treatment based on selectively targeting the ubiquitin-proteasome-machinery.

Really Interesting New Gene (RING) finger variants of zinc finger motifs act as ubiquitin E3-ligases and target proteins including p53 to the proteasome [33]. Since PHD and RING finger motifs are both forms of zinc fingers, it was speculated that some PHDs also act as ubiquitin E3-ligases [34], but closer inspection of PHD regions did not confirm this hypothesis [35].

Based on this background, and a previous study indicating that INGs physically interact with at least 16 proteins directly involved with proteasomal degradation such as regulatory subunits of both the 20S and 26S-proteasome [8], we asked *a)* whether ING1 stabilizes p53, and if so, *b)* whether ING might do this through affecting ubiquitin metabolism, thereby shielding p53 from proteasomal degradation.

We discovered a region adjacent to the PHD of ING1 that acts as a ubiquitin-binding domain (UBD). We also found that ubiquitin competes with PI signaling lipids for ING1 binding and that physiological levels of ING1 stabilize monoubiquitinated forms of the p53 tumor suppressor via its UBD. We also provide data regarding the mechanism by which the ING1 type II tumor suppressor stabilizes p53 through a pathway involving the localization of the herpesvirus-associated ubiquitin-specific protease (HAUSP), a p53 and MDM2 deubiquitinase (DUB). These findings could account for the frequently reported activation of p53 as an inducer of apoptosis by the ING proteins and directly link lipid stress signaling to ubiquitin-mediated proteosomal degradation through competition for the polybasic regions found in ING family proteins.

## Results

### ING1 expression stabilizes p53

**Figure 1:**
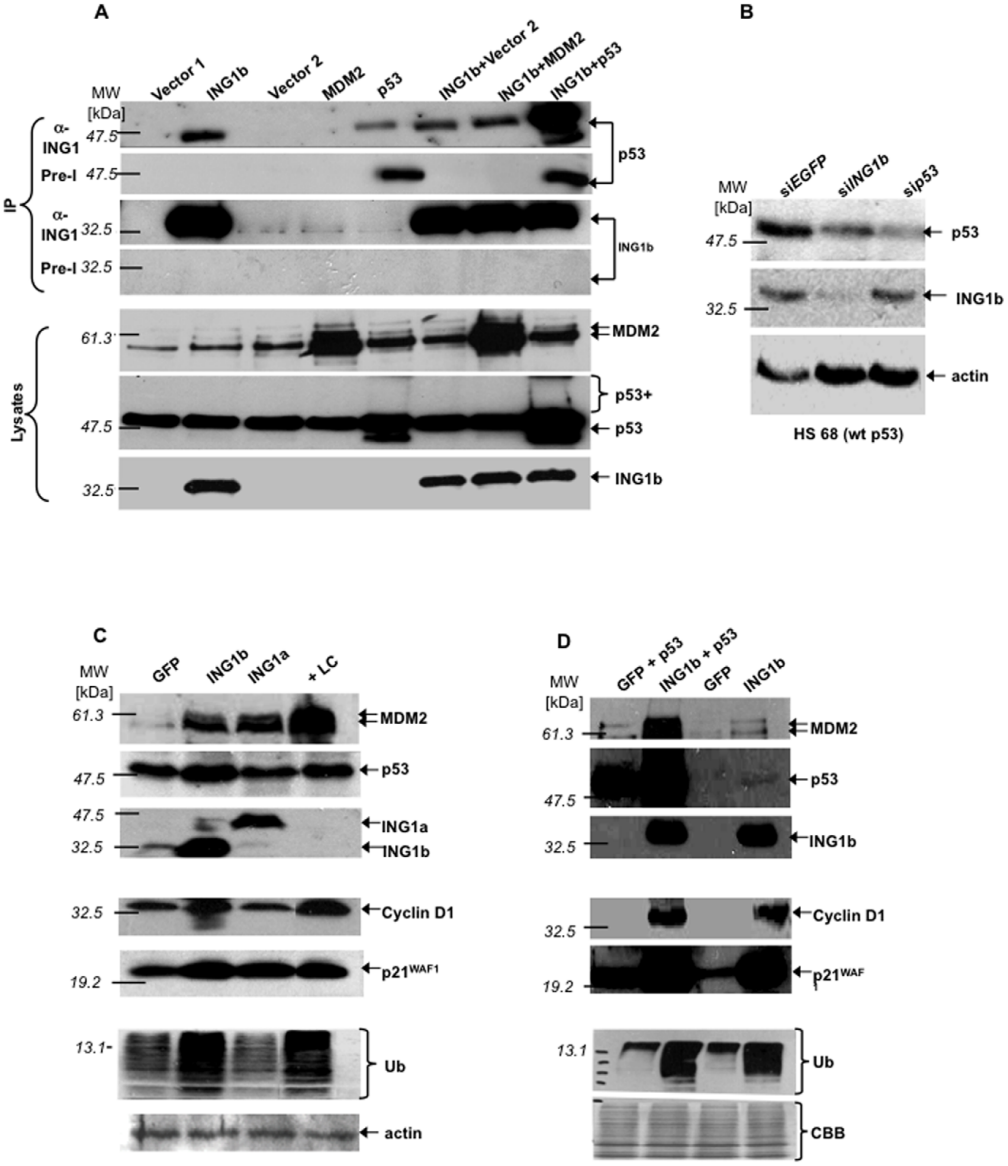
ING1 stabilizes p53. ***A*** *)* Lysates from 293 cells transfected with the indicated constructs were immunoprecipitated (IP) with preimmune (Pre-I) or α-ING1 and blotted with the indicated antibodies. Bottom panels confirm protein expression. ***B*** *)* Hs68 cells stably transfected with si*EGFP*, si*ING1b* or si*TP53* were analyzed for the indicated proteins. ***C*** *) Lysates from Hs68 or* ***D*** *)* from 293 cells transfected with the constructs indicated or treated with lactacystin (+LC) were blotted with indicated antibodies. Ubiquitin (Ub) visualized by two antibodies gave similar results. MDM2 antibodies typically detected two polypeptides. *Abbreviation: CBB-*Coomassie-stained loading-controls.

Pilot studies indicated that at levels of sensitivity where physical interactions were seen between endogenous ING1 and other proteins [36], those between ING1 and p53 were only observed when both proteins were overexpressed. Increasing assay-sensitivity in this study revealed that p53 was specifically recovered in ING1-immunoprecipitates (IPs) when ING1 was overexpressed, while p53-overexpression resulted in recovery of p53 in both α-ING1 and nonspecific preimmune-IPs (Figure 1A). Co-expression of MDM2 and ING1 did not alter p53-levels recovered in ING1-IPs compared to expressing ING1 alone, suggesting that ING1 does not compete with MDM2 for p53-binding and so MDM2 does not affect ING1-induced p53 stabilization in this assay.

After co-expression of p53 and ING1, high p53-levels were recovered in ING1-IPs. This included higher molecular weight isoforms of p53 (Figure 1A: “p53 +”), possibly representing increased levels of post-translationally modified forms of p53. RT-PCR showed that ING1 did not increase *p53* transcript-levels (supplementary figure S1). Furthermore, examination of cells injected with GFP, or GFP and ING1 constructs showed that ING1 altered nuclear and nucleolar morphology and increased p53-levels in both nucleus and cytoplasm, primarily in a perinucleolar pattern (supplementary Figure S2), consistent with INGs affecting p53 levels [19], [37].

### *ING1* stabilizes *p53* at endogenous levels

We next down-regulated *ING1* and *p53* using siRNA. Compared to the control (si*EGFP*), si*ING1* lines showed considerably lower ING1-levels and a remarkable decrease in p53-levels, confirming that at endogenous levels, ING1 stabilizes p53. In contrast, *p53*-knockdown (si*p53*) did not affect ING1-levels (Figure 1B), nor did knocking down *ING1* alter p53-levels in cells with high p53-expression, such as HEK-293 cells containing HPV E6-stabilized p53 (supplementary Figure S3).

### ING1 stabilizes other proteins degraded by proteasome pathways

To investigate whether ING1 affected levels of other proteins regulated by the ubiquitin-mediated proteasome pathway, primary human Hs68 fibroblasts were transfected with the two major ING1 splicing isoforms, ING1A and ING1b, or treated with the proteasome-inhibitor lactacystin: ING1b stabilized p53, p21^WAF1^ and cyclin D1 as effectively as lactacystin, and MDM2 to a lesser degree, while ING1a stabilized p21^WAF1^ and MDM2, but not p53 or cyclin D1 (Figure 1C). These results are consistent with reports that ING1b, but not ING1a, collaborates with p53 in biological assays [37], and that ING1b induces apoptosis while ING1a induces senescence [5]. Blotting with α-ubiquitin (Ub) showed that ING1b increased levels of a wider variety of ubiquitinated proteins than ING1a, exerting effects similar to lactacystin (Figure 1C).

To test if stabilization of p53 was due to altered stoichiometry as a consequence of ING1-overexpression, ING1b and p53 were co-expressed (Figure 1D). ING1b-overexpression stabilized high levels of ectopically expressed wild-type (wt)-p53 and cyclin D1 in the absence or presence of overexpressed p53, while p21^WAF1^ was slightly higher when both ING1b and p53 were overexpressed. This is expected since p53 induces *P21^WAF1^*-transcription and ING1b stabilized both p21^WAF1^ and p53. Similarly, MDM2 was accumulated to a much higher degree when ING1b and p53 were co-expressed, since it is also transcriptionally induced by p53. Taken together, ING1b-overexpression increased the levels of many ubiquitinated proteins. To confirm this effect by an independent method, cells overexpressing ING1 were stained for ING1 and Ub: Cells expressing higher levels of ING1 show markedly elevated levels of Ub (supplementary figure S4).

### ING1 stabilizes ubiquitinated forms of p53

**Figure 2:**
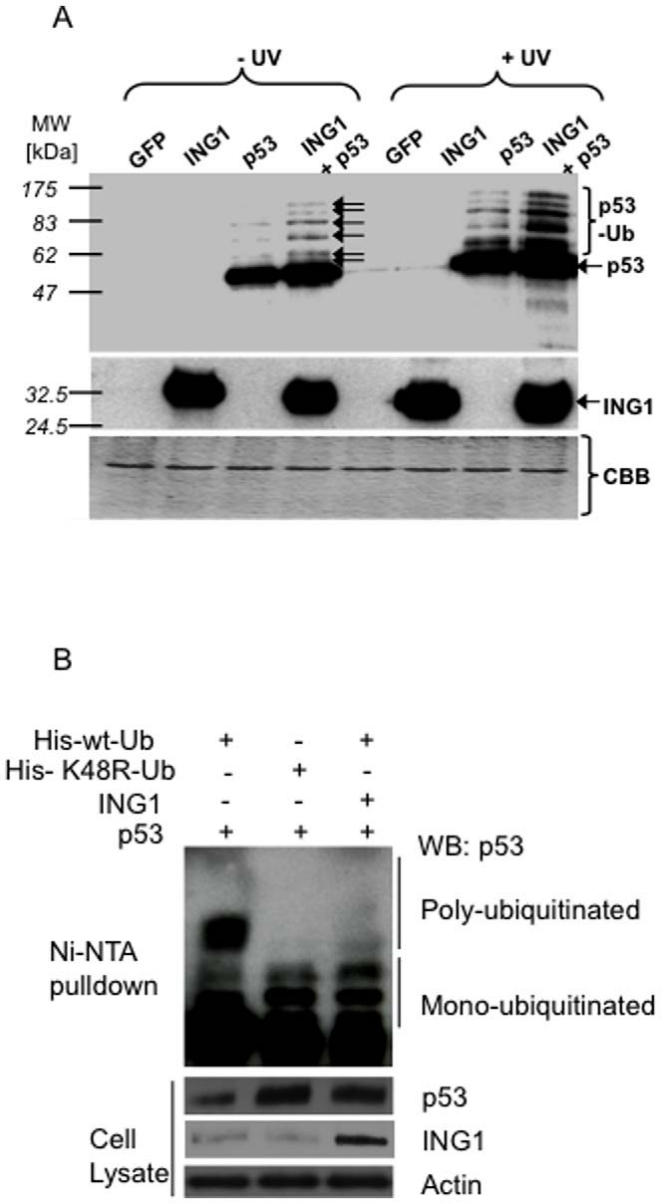
ING1 stabilizes ubiquitinated forms of p53. ***A*** *)* Lysates of Hs68 cells infected with the constructs indicated were exposed to UV (25 J/m^2^) and blotted with α-p53. *Arrows*: ubiquitin-conjugated forms of p53 (p53-Ub) induced by UV and stabilized by ING1b expression. Lower panels confirm ING1b expression and protein loading (CBB). ***B*** *)* HEK293 cells were transfected with the expression constructs indicated and grown for 24 hours. Cells were then lysed, lysates were clarified by centrifugation and lysates were incubated with Nickle (Ni)-NTA agarose beads. Beads were washed, eluted with imidazole and eluates were resolved using SDS-PAGE. After transfer to membranes p53 protein was detected by western blotting (WB). The input samples from cell lysates that were used for the pull downs confirm that equal amounts of lysates were used with the actin loading control and also were used to determine the levels of ING1 and p53.

To test whether ING1 blocked polyubiquitin-mediated degradation, cells transfected with GFP, GFP and ING1, GFP and p53 or GFP and ING1 and p53 were left untreated or treated with UV, and lysates were blotted for p53. UV increased p53-levels, particularly of several p53-variants with lower electrophoretic mobility (Figure 2A). These variants were of the same mobility as ones further increased in response to ING1-overexpression. They could represent p53 with variable numbers of monomeric ubiquitin-moieties bound to a subset of the 20 potential target lysine-residues of p53 or polyubiquitinated forms of p53. Six of these 20 lysines are targeted by the MDM2-Ub-ligase which monoubiquitinates p53 [29], [30], and six modified forms of p53 were observed in response to UV and ING1-overexpression (arrows in Figure 2A). The mobility of the slowest isoform corresponds to ∼100 kDa, consistent with p53 having six ubiquitin-moieties of 8.541 kDa bound to the six known target-residues.

To further test the nature of these modified forms of p53, we compared the multiple bands observed in cells expressing p53 and ING1 with the p53 forms observed in cells expressing a K48R-Ub mutant that inhibits poly-ubiquitination of p53, leading to accumulation of multi-monoubiquitinated proteins that appear as higher molecular weight forms in SDS-PAGE. His-tagged wt or K48R mutant Ub plasmid was co-transfected with p53 and ING1b and ubiquitinated proteins were pulled down using Nickle (Ni)-NTA agarose beads. The ubiquitinated forms of p53 were detected by western blotting (WB) (Figure 2B). Cells expressing either ING1b or K48R-Ub showed very similar bands for p53, while cells transfected with wt-Ub displayed additional lower mobility forms of p53 indicative of polyubiquitination. Furthermore, expression of both mutant Ub and ING1b led to increased levels of unmodified p53 compared to wt-Ub expressing cells (Figure 2B). This observation further supports the contention that ING1 acts to prevent the formation of polyubiquitinated forms of p53, resulting in the accumulation of multimonoubiquitinated and unubiquitinated forms.

Transfection of *ING1* increased p53-levels in cells with wt-, but not with mutant p53 (supplementary Figure S5). Scanning of blots and ELISA experiments indicated that ING1b, but not ING1a, stabilized p53 and increased the overall levels of ubiquitinated proteins by about three-fold, compared to about four-fold in response to lactacystin (supplementary Figure S6). To ask if ING1 binds and stabilizes p53 in part via binding Ub, pulldown assays were performed. ING1b, but not ING1a or p53, bound Ub-agarose beads (supplementary Figure S7). Binding was specific since ING1b did not bind agarose bead negative controls. Re-probing showed that p53 was also recovered by Ub-agarose beads, but only in cells overexpressing ING1b. This indicates the formation of Ub-ING1b-p53-complexes, since p53 was not seen in the absence of ING1b-overexpression.

**Figure 3:**
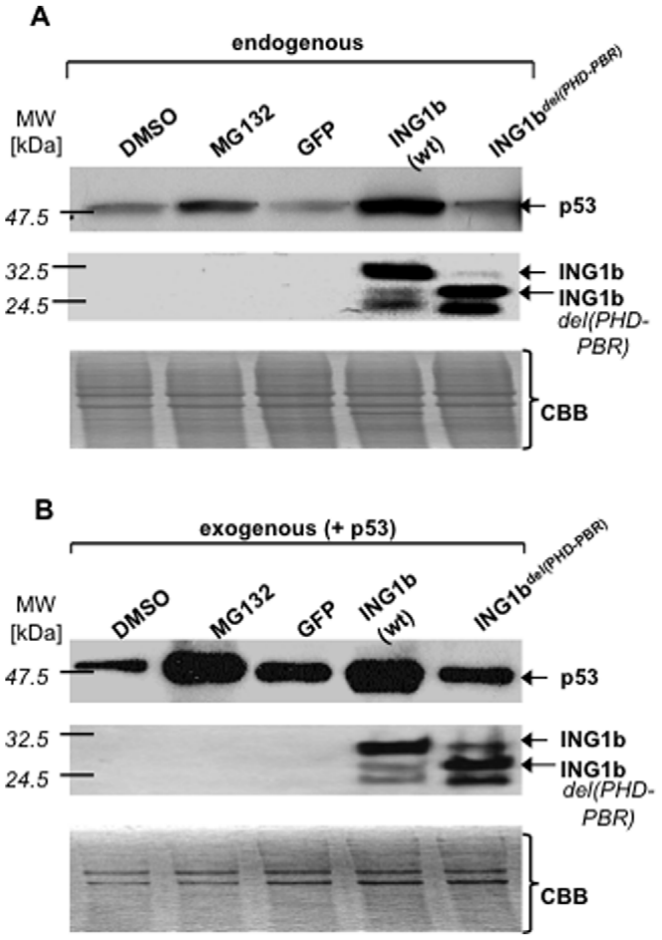
Discovery of a region adjacent to the *ING1b*-plant homeodomain (PHD) that acts as a ubiquitin-binding domain (UBD). ***A*** *)* Lysates from Hs68 cells treated or transfected as indicated and blotted for p53. Lower panels confirm ING1-expression and loading. ***B*** *)* Cells treated as in *A)*, but also co-infected with *p53-*adenovirus, were blotted as in *A*). *Abbreviations:* CBB: Coomassie-stained loading-controls; *GFP:* green fluorescent protein, *ING1b* ^del(PHD-PBR)^: *ING1b*-construct with PHD-PBR deletion, *PBR:* polybasic region, *wt*-wild-type.

Given that the ING2-PHD was required for activating p53 [17], we next examined if an *ING1-*carboxyl-terminal deletion (removing the PHD and adjacent PBR) stabilized unmodified and/or monoubiquitinated p53. Wt-, but not the deleted form of ING1 stabilized both endogenous (Figure 3A) and ectopically expressed p53 (Figure 3B) to a degree comparable to the effect of the proteasome-inhibitor MG132.

### ING1 contains a ubiquitin-binding domain (UBD) adjacent to its PHD

Since ING1 promoted accumulation of ubiquitinated forms of p53, we examined the ING1 protein sequence for motifs known to be involved in Ub-binding [38]. We identified a UBD adjacent to the ING1 PHD (supplementary Figure S8), which was previously described as a PBR, necessary and sufficient for the binding of PIs [17]. Nuclear magnetic resonance (NMR) analysis has shown that UBD binding can block access to the K48 residue of Ub, thereby blocking polyubiquitination that targets proteins to the proteasome [39]. Given that several proteins affecting proteasomal pathways contain UBDs, this suggested a role for ING1 in regulating p53 stability through this pathway.

### ING1 does not stabilize p53-mutants lacking sites of ubiquitination

**Figure 4:**
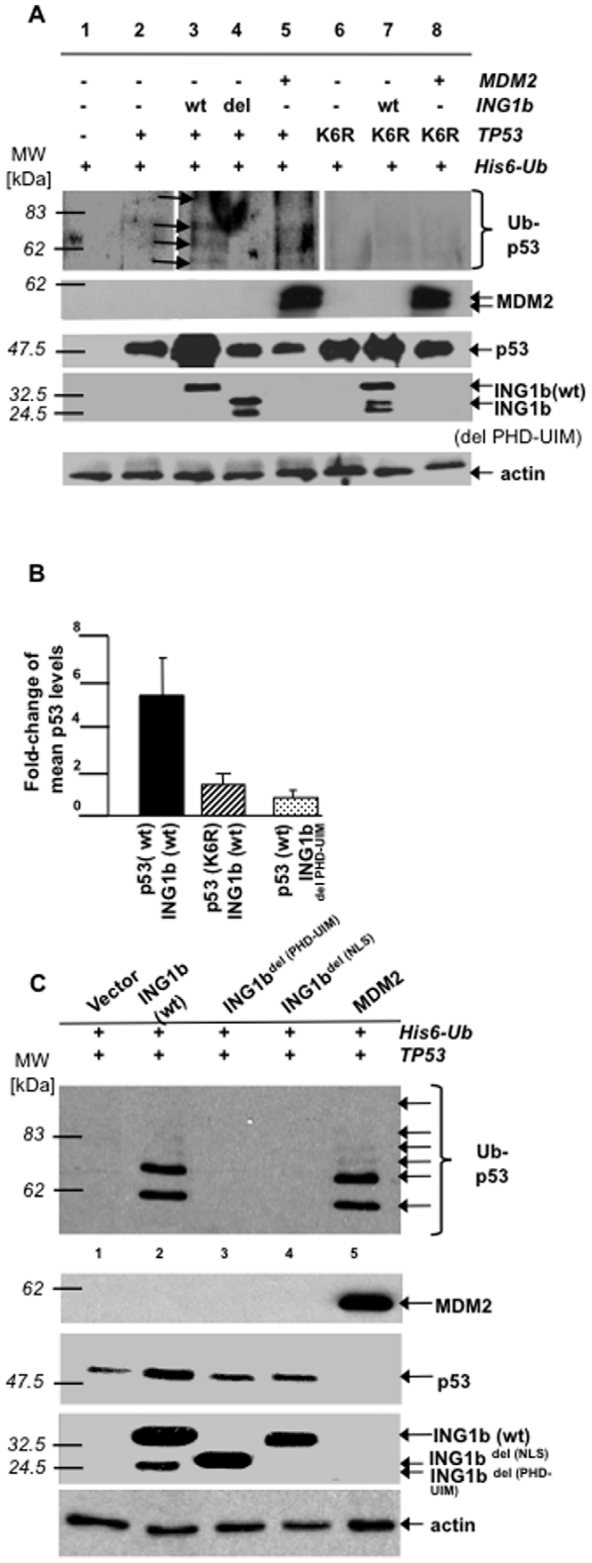
*ING1b* does not stabilize p53-mutants lacking sites of ubiquitination. ***A*** *)* H1299 cells transfected as indicated were blotted for p53 following nickel-agarose-precipitation and *in vivo* ubiquitination. *Arrows:* ubiquitinated species of p53 (Ub-p53). Lower four panels confirm indicated protein-expression and protein loading (actin). ***B*** *)* Fold-changes of mean p53 levels after co-expression of indicated forms of p53 and ING1b (three independent assays scanned by densitometry). *Error bars:* standard deviations. ***C*** *)* H1299 cells were co-transfected as indicated and lysates immunoprecipitated using p53-antibody linked to Protein-A-agarose. Precipitates were blotted using α-His. Lower four panels confirm p53-, MDM2- and ING1b-expression and protein loading (actin). *Abbreviations: ING1b* ^del(PHD-PBR)^: *ING1b*-construct with PHD-PBR-deletion; *ING1b* ^del(NLS)^: *ING1b*-construct with NLS-deletion; *ING1b* ^del(PHD-UIM)^: *ING1b*-construct with PHD-UIM-deletion; p53(K6R): p53 containing six ubiquitinated lysine-residues that are targets for ubiquitination (Lys367, Lys369, Lys370, Lys378, Lys379, and Lys383) mutated to arginine; NLS: nuclear localization-sequence; PBR: polybasic region; PHD: plant-homeodomain; UIM: ubiquitin-interacting motif; wt: wild-type.

To ask if ING1-mediated stabilization of p53 was Ub-dependent, a Histidine (His)6-Ub construct was co-expressed in H1299 (*p53*-/-) cells with wt- and mutant forms of ING1, and with wt- and mutant *p53* (“p53K_6_R” containing six lysine residues, that are targets of ubiquitination [39], mutated to arginine). Wt-*ING1* and MDM2, but not ING1 lacking the PHD and UBD (ING1b^del(PHD-UBD)^), promoted the formation and/or stabilization of similar ubiquitinated forms of p53 (arrows in Figure 4A). In contrast, p53K_6_R was not affected by ING1 or MDM2. Wt-, but not mutant ING1 stabilized wt-p53, but not p53K_6_R, by several-fold (Figure 4A, B).

### The PHD and UBD of ING1 stabilize forms of modified p53 similar to those produced by MDM2

IP-Western analysis showed that wt-, but not ING1b^del(PHD-UBD)^ stabilized the same six ubiquitinated forms of p53 as produced by the MDM2 Ub-E3 ligase (Figure 4C, top panel, lanes 2,5). An ING1-mutant lacking the PHD, but containing an intact UBD, gave only partial stabilization of p53 suggesting a contextual requirement for UBD-activity as previously reported for other UBDs [38]. While corroborating nickle-agarose experiments, this assay favored recovery of p53 with one or two Ub-moieties. This may be due to multiple monoubiquitination interfering with the binding of the p53 monoclonal antibody used, since both the ubiquitination sites and the antibody recognition site are located in the carboxyl region of p53.

Wt-ING1 stabilized both ubiquitinated and non-ubiquitinated forms of p53 (Figure 4C, lane 2), while MDM2 only increased levels of p53 monoubiquitinated on several residues (Figure 4C, top panel, lane 5), but failed to promote accumulation of non-ubiquitinated p53 (Figure 4C, third panel, lane 5). This implies that MDM2 promoted multiple monoubiquitination of p53 with subsequent polyubiquitination of residues by p300 resulting in proteasomal degradation [28], while ING1 promoted stabilization of both monoubiquitinated and non-ubiquitinated forms of p53, consistent with data in Figure 2B. Furthermore, while the UBD seemed to be primarily responsible for interacting with Ub, binding to unmodified forms of p53 was dependent on the function of at least three regions of ING1: the NLS, the PHD and the UBD (Figure 4C, lanes 3,4) - regions flanking the 14-3-3 binding site on serine 199 of ING1 that promotes 14-3-3-mediated export of the ING1 protein to the cytoplasm [40]. Consistent with these data, co-expression of wt- and PHD-UBD-deleted forms of ING1 with wt-p53, or with the p53K_6_R-mutant, followed by IP-western-analysis using ING1 antibodies, revealed a strong interaction of wt-ING1 with wt-p53 and a weak one with p53K_6_R (supplementary Figure S9). Blotting for ING1 and p53 showed that higher levels of mutant ING1 were expressed revealing the difference in binding affinity even more.

ING1 bound ubiquitinated forms of p53 through its PHD-UBD region and induced the accumulation of similar ubiquitinated forms of p53 as did UV (Figure 2A), expression of ubiquitin K48R (Figure 2B), the proteasome-inhibitor MG132 (Figure 3A, B) and MDM2 (Figure 4A), suggesting that it stabilized p53 that was monoubiquitinated on particular lysine residues. If so, then mutant p53, that could not be ubiquitinated on these residues, while being inherently stable, would not be further stabilized by ING1, which was the case in this study (lower panels of supplementary Figure S9, lanes 1 and 2 compared to lanes 4 and 5).

### ING1 binds and acts through HAUSP to affect p53-levels

**Figure 5:**
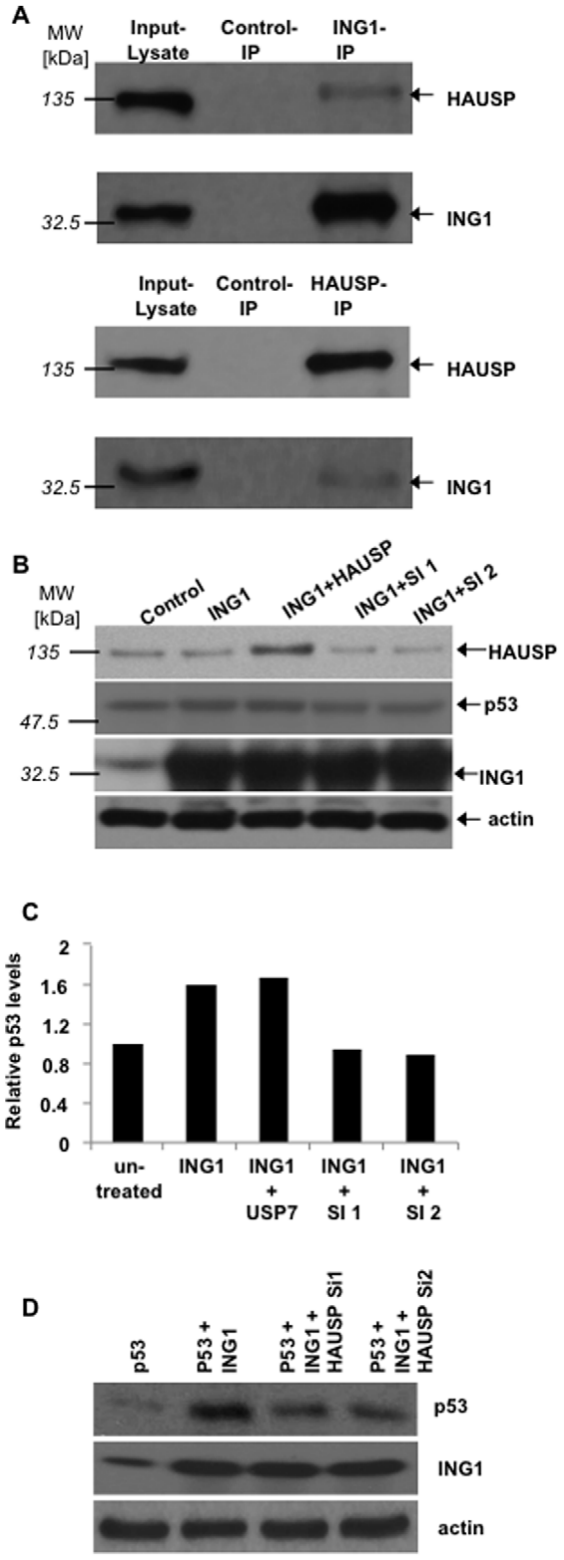
ING1 binds and acts through the herpesvirus-associated ubiquitin-specific protease (HAUSP) to affect p53-levels. ***A*** *)* HEK293 cells were harvested and immunoprecipitated (IP) as indicated and with controls (rabbit pre-immune sera for HAUSP or mouse anti-GST for ING1). IPs were blotted for HAUSP (USP7) or ING1. ***B*** *)* HEK293 cells were transfected as indicated, including two different *HAUSP*-siRNAs (Si1, Si2). **C** *)* Results from experiments such as the one shown in B) were scanned by densitometry. *Columns:* average of two independent values with p53-levels in untransformed cells set to 1.0. Relative p53-values represent levels seen in both transfected and untransfected cells in panels B) and C). **D** *)* HEK293 cells cotransfected with p53 and HAUSP in the absence and presence of two different HAUSP siRNAs.

Several Ub-E3 ligases and deubiquitinases (DUBs) can affect p53 stability, and HAUSP (also known as USP7) can bind to and affect the stability of both MDM2 and p53 [30]. To identify the different potential regulators of p53-activity affected by ING1, ING1-IPs were examined for the presence of HAUSP: Endogenously expressed HAUSP was indeed recovered in ING1-immunoprecipitates and the reciprocal IP-western confirmed their interaction (Figure 5A). If such interaction served to target HAUSP to p53 and retain it in a non-polyubiquitinated state, then HAUSP should be necessary for stabilization of p53 by ING1. To test this idea, ING1 was transfected into cells in the presence of HAUSP expression constructs or two different HAUSP siRNAs. As shown in Figure 5B, cells expressing ING1 showed higher p53-levels, cotransfection with HAUSP slightly increased this effect while two different siRNAs targeting HAUSP completely blocked the ability of ING1 to stabilize endogenous p53. The average p53-levels from two independent experiments under these conditions are shown in Figure 5C. Similar results, but of a greater magnitude were observed with overexpressed p53 in HEK293 cells as shown in Figure 5D. The absolute degree of p53-increase in response to ING1 was not as great as seen in previous experiments, since these data reflect a more modest transfection efficiency. Nevertheless, cotransfection of ING1 with both siRNA-species would only detect transfected cells and showed complete blockage of ING1-induced p53 stabilization.

### Ubiquitin competes with PIs for binding the ING1-UBD

**Figure 6:**
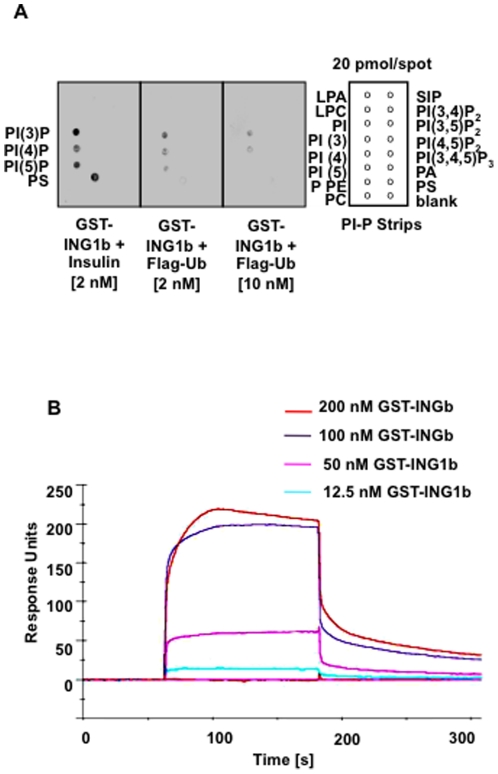
The *ING1b* carboxyl region competitively binds phospholipids and ubiquitin. ***A***) Phosphatidylinositol-phosphate (PI-P) strips containing lysophosphatidic acid (LPA), lysophosphocholine (LPC), phosphatidylinositol (PI), PI(3)P, PI(4)P, PI(5)P, phosphatidylethanolamine (PE), phosphatidylcholine (PC), sphingosine 1-phosphate (S1P), PI(3,4)P_2_, PI(3,5)P_2_, PI(4,5)P_2_, PI(3,4,5)P_3_, phosphatidic acid (PA), phosphatidylserine (PS), and a blank spot in the shown configuration. Purified GST-ING1b plus insulin (negative control) or Flag-ubiquitin with the concentrations indicated were co-incubated with PI-P-strips and protein-lipid blot assays were performed to detect bound GST-ING1b using α-ING1. Three independent experiments gave similar results. ***B*** *)* Surface plasmon resonance analyses of full-length GST-ING1b binding to immobilized FLAG-ubiquitin.

Since the UBD we identified was recently described as a PBR that was necessary and sufficient for binding of PIs [17], we asked whether Ub competes for ING1-binding with monophosphorylated lipid species previously shown to bind ING2 [17], [41]. We found that full-length ING1 had a marked preference for binding the same phospholipids (PI(3)P, PI(4)P, PI(5)P) as previously reported for the isolated PBR of ING2 [17] (Figure 6A). Also, Ub showed dose-dependent inhibition of ING1b-binding to lipids. That was not observed with control peptides (insulin). Results from three independent surface plasmon-resonance experiments gave a peptide dissociation constant (K_d_) for Ub-ING1b-binding of ∼100 nM (Figure 6B), suggesting slightly more avid binding than reported for ING-PHD-histone H3 peptide interactions that varied from 1.5 µM to 2.3 mM depending on the histone methylation state [12].

## Discussion

In this study, we identified the PBR adjacent to the ING1-PHD as a novel UBD. We also showed that the PHD and UBD of ING1 stabilize the same forms of p53 that are stabilized by DNA-damage or by proteasome-inhibitors. These also co-migrate with monoubiquitinated forms of p53, generation of which by the Ub-E3 ligase MDM2 results in relocalization of p53 rather than proteasomal degradation [29], [30]. Based on these data and the significant role of proteins with UBDs in various processes such as the DNA-damage-response [39], this study suggests a role for ING1 in increasing the proapoptotic functions of p53, and thus a new model of stress-induced p53-activation.

**Figure 7:**
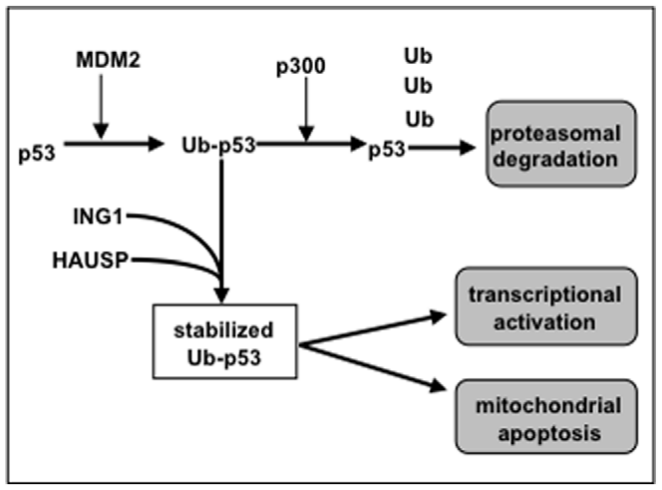
A model for the stabilization and activation of p53 by *ING1b.* Data presented in this study support a model in which p53 is continually expressed, monoubiquitinated on up to six different lysine residues and then is rapidly targeted for proteosomal degradation by polyubiquitinization through K48 residues by the p300/E3-ligase. Under conditions of stress ING1 is relocalized by binding phosphatidylinositol-5 monophosphate which can then be replaced by binding ubiquitin (Ub) located on p53. ING1 either imports, or subsequently binds herpesvirus-associated ubiquitin-specific protease (HAUSP) to prevent or reverse the polyubiquitination of mono- or multimonoubiquitinated forms of p53, thereby stabilizing these and also unubiquitinated forms of p53 to promote either transcriptional activation or mitochondrial-mediated apoptosis.

In this model (Figure 7), non-ubiquitinated p53 is produced continually and monoubiquitinated on multiple lysine-residues by MDM2. The p300/E4-ligase then elongates Ub-chains and targets p53 to the proteasome [28]. UV and other stresses induce ING1b-binding to p53 in an Ub-facilitated manner, helping to target ING1-associated HAUSP to p53, thereby stabilizing p53 due to HAUSP-mediated deubiquitination of nascent polyubiquitin chains. Co-localization of ING1 and p53 also promotes acetylation of p53 (p53ac) by ING on lysine-residue 382 [19], [42], which subsequently activates p53 as a transcription factor. UV also induces the formation of bioactive stress-signaling PIs [42], [43] that bind ING1 and ING2 on a site overlaping the Ub-binding-site [17]. PIs may subsequently competitively displace Ub and trigger the release of free p53 at high local concentrations that favor multimerization to induce p53-DNA-binding [44]. ING1-bound monoubiquitinated p53 could also be transported to the cytoplasm via 14-3-3-mediated cytoplasmic relocalization of ING1 [40], where p53 directly affects mitochondria-based apoptosis [45]-[48].

While this model predicts that ING1 stabilizes p53, no induction or stabilization of *ING1* mRNA or ING1-protein by p53 would be predicted, as noted (supplementary Figure S1) and previously reported [49]. This model is supported by the competition between PIs and Ub for ING1b-binding, providing direct evidence that INGs can link stress-induced PI-signaling to Ub-mediated protein metabolism. It also indicates that ING1b-mediated stabilization and translocation of p53 to the cytoplasm and subsequently to the mitochondria, but not activation of nuclear p53 transcriptional activity, is one of the mechanisms by which ING proteins might potentiate p53-mediated apoptosis. Identifying the individual contributions of ING1-binding to the proteins listed at http://www.visualgenomics.ca/gordonp/S2.html [8] that are known to regulate proteasomal activity will further clarify the basis of Ub-dependent stabilization of p53.

Given that *ING1* is down-regulated in many human malignancies [2], [3], this model implies ING1 as a prognostic factor for response to targeted cancer therapy: patients with tumors expressing low ING1-levels might not significantly profit from certain proteasome-inhibitors or other anticancer agents, that involve the induction of p53-dependent apoptosis in the selective killing of tumor cells.

## Materials and Methods

### Cell culture

Hs68 (ATCC CRL-1635; wild-type (wt)-*TP53*), 293 (ATCC CRL-10852) and H1299 cells (ATCC CRL-5803; *TP53*-/-) were obtained from ATCC. U251 (mutant *TP53*) and HCT116 (wt-*TP53*) cells were from P. Forsyth and B. Vogelstein, respectively. Hs68, 293, and U251 cells were grown as described [50]. HCT116 cells were grown in McCoy's 5a with 1.5 mM L-glutamine, 10% FCS. H1299 cells grew in RPMI containing 10% FBS. Cells were maintained at 37°C in 95% air and 5% CO_2_. UV-treatment was done in the absence of medium after which media, in which pre-exposed cells were growing, were replaced. All cell lines and strains tested negative for mycoplasma.

### DNA constructs, transfection and infection

*ING1a, ING1b*, *ING2* and *TP53* were subcloned into pAdTrack-CMV containing a separate *EGFP* expression cassette and recombined with “pADEasy”-1 in BJ5183-*E. coli*. Recombinants were re-amplified in XL-Blue, linearized (PacI) and transfected into 293 cells for packaging. Viral clones were selected for expression, plaque amplified and purified over CsCl_2_. The *ING1*, *TP53* and *MDM2* genes were cloned in pCI (Clonetech). *ING1* deletions were generated by one-step-PCR. Optimized adenoviral infections were done at multiplicities of infection (MOI) of 100 for Hs68, 20–50 for U251 and HCT116 cells giving ∼95% infectivity as monitored by green fluorescent protein (GFP) expression. No toxicity was observed when adenoviruses were used at the MOIs indicated. Transient transfections of plasmids were performed using a modified calcium phosphate method for 293 and H1299 cells.

### Small interfering (si)RNA

Multiple splicing isoform-specific siRNAs to *ING1b* and *EGFP* (negative control) were designed and cloned into psilencerU6 (Ambion). Constructs were used to generate stable lines in HCT116 cells. After multiple passages, lines were tested for gene expression using RT-PCR primers (sequences available upon request) for *p53*, *ING1* and control RNAs (*GAPDH* or *β-actin*).

### Microinjection and indirect immunofluorescence

Hs68 cells synchronized in G_1_ were microinjected as described [50]. Constructs encoded GFP or GFP and ING1. Cells were fixed 24h after injection and stained for p53 with a mixture of DO1, PAb240 and PAb421 monoclonal antibodies and with secondary Cy-3 fluorescent antibodies (Amersham) for 1h at 37°C.

### Western and immunoprecipitation (IP)–western assays

Cells were infected or transfected with the adenoviral constructs or plasmids encoding p53, ING1, MDM2 and two vector controls. Cells were treated with 8 mM lactacystin for 6h, with 30 µM MG132 for 4 h or with 15 µM MG132 for 16 h (Calbiochem) as indicated. After cell lysis, samples were blotted with anti(α)-p53 (Santa Cruz-6243), α-cyclin D1 (Cell Signalling-2926) or α-cyclin A polyclonal (Santa Cruz-751), α-MDM2 (Santa Cruz-812) or α-ubiquitin (α-Ub) monoclonal (Santa Cruz-9133), α-Histidine (α-His-6) (Santa Cruz-803), α-p21^WAF1^ polyclonal (Santa Cruz-397), α-actin polyclonal, α-p27 monoclonal, α-herpesvirus-associated ubiquitin-specific protease (α-HAUSP) (Bethyl Laboratories), α-ING1 monoclonal and α-ING1b isoform-specific polyclonal antibodies (Southern Alberta Cancer Research Institute (SACRI) Antibody Services). Information regarding the particular epitope(s) recognized by the different ING1 antibodies is provided in Suzuki et al., 2011 [51]. In UV-experiments, Hs68 cells were exposed to 30 J/m^2^ of UV 18 h post-infection and harvested 12 h after UV. IP-westerns were done as described [11].

### RT-PCR

Hs68 cells were harvested 24 h post-infection and extracted for RNA using Trizol (Invitrogen). DNAse treatment preceded confirmation of RNA quality by absorbance, visual inspection (1% denaturating agarose gel electrophoresis and EthBr staining) and RT-PCR for *GAPDH*. cDNA samples were used to estimate relative *TP53-*, *P21^WAF1^*-, *MDM2-*, *CyclinD1-* and *ING1*-levels by comparison to internal controls (*GAPDH, β-actin*) (primer-sequences available upon request). Aliquots of PCR products equalized to give equivalent signals from the internal control mRNAs were electrophoresed through 2% agarose gels (Pharmacia), stained with 0.2 mg/ml EthBr, analyzed by computerized densitometric scanning of the images (Kodak imaging software) and normalized using internal controls.

### Sequence analyses

All sequences used were from NCBI and multiple alignments were done (EMBL Clustal-W-program) (sequence accession numbers available upon request).

### Ubiquitin (Ub) pull-down assays

HCT116 cells infected with adenovirus or transfected with plasmids were harvested at +24 h and lysed in NP-40-buffer. Lysates were incubated with Ub-agarose (Biochem) or Protein A-agarose beads (Pharmacia; negative control) for 4 h at 4°C. Beads were washed briefly and proteins were solubilized in SDS-buffer, electrophoresed and blotted with α-ING1 monoclonals or α-p53 polyclonal antibodies. To analyze p53 ubiquitination, HEK293 cells were transfected with plasmids encoding p53, ING1, His-wt-Ub and His-K48R Ub and grown for 24 hours. They were then lysed and the cleared lysate incubated with Nickle (Ni)-NTA agarose beads (Qiagen) for one hour. The beads were then washed and proteins eluted with 500 mM imidazole. They were then resolved using SDS-PAGE and p53 was detected by western blotting.

### *In vivo* ubiquitination assay

H1299 cells were transfected with 1 µg wt-*p53* or *p53-*K_6_R constructs or with 2 µg each of the different *INGs*, *MDM2* (positive control), and His6-tagged-*Ub*. Purification of His-6-ubiquitinated conjugates was done as described [29]. 36 h after transfection, cells were washed twice with PBS and harvested. 20% of each suspension was used for protein staining and western blots, the rest for isolation of ubiquitinated conjugates.

### Lipid binding and lipid-ubiquitin competition assays

Protein-lipid blot assays were done as described [43]. For competition assays, the indicated control (insulin) or competitor (ubiquitin) polypeptides were incubated with GST-tagged affinity purified ING1 prior to incubation with phosphatidylinositol-phosphate (PIP) strips (Echelon Bioscience) at 4°C . Bound ING1 was visualized using α-GST.

### Surface plasmon resonance (SPR)

Binding experiments were done by the SACRI Antibody Facility on a Biacor 3000 biosensor at 25°C. Recombinant FLAG-ubiquitin (Boston Biochem) was immobilized on CM-5 chips by amine cross-linking, increasing amounts of recombinant GST-ING1 were injected at 30 ml/min and SPR-response was recorded. Sensograms were obtained by subtracting signals from the reference surface (blank) from the signal obtained from the FLAG-ubiquitin surface. Binding assays were repeated thrice. After curve fitting and averaging, results were obtained from both steady-state affinity and kinetic data.

## Supporting Information

Figure S1 RT-PCR using *GAPDH* or *actin* as internal amplification controls was performed to estimate *TP53-*, *P21* ^WAF1^-, *ING1b-*, *MDM2-*, or *Cyclin D1*-mRNA-levels in Hs68 fibroblasts transfected with the indicated constructs. *Bar-graph*: mean-mRNA-levels of three independent experiments, setting green fluorescence (*GFP*; negative control) to zero. *Error bars:* standard deviations.
(TIF)

Figure S2 Hs68 fibroblasts injected with the indicated constructs were stained for DNA (DAPI) and p53. Green fluorescence (GFP) identifies injected cells. *Arrows*: nucleolar morphology in the absence and presence of elevated ING1b. p53 levels were elevated in 55 of 60 cells examined and localization was nuclear, perinuclear and cytoplasmatic. Bar  =  10 µm.
(TIF)

Figure S3 HEK293 cells transfected with the indicated siRNAs were analyzed for p53, ING1b and actin levels.
(TIF)

Figure S4 *ING1b*-transfected Hs68 cells were stained for DNA (DAPI), ING1 and ubiquitin (Ub). The arrow highlights a transfected cell.
(TIF)

Figure S5 Lysates from cells with wild-type (wt; Hs68) or mutant (U251; containing the p53R273H mutation) p53 infected with the indicated constructs were blotted for p53. *CBB-lanes*: Coomassie-stained loading controls. Green fluorescence (GFP) indicated infection efficiencies of >95% in all cases.
(TIF)

Figure S6 Dot-blot dilution series of lysates from Hs68 fibroblasts treated for 12h with lactacystin (+LC) or left untreated (-LC) served as positive and negative controls, respectively, for lysates from cells infected with the adenoviral constructs indicated. Blots were probed with ubiquitin (Ub) antibodies. Signals from three independent ELISA experiments using Hs68 fibroblasts were quantitated by scanning densitometry and plotted. *Error bars*: standard deviations.
(TIF)

Figure S7 Lysates of HCT116 cells (*TP53* -|-) infected with the indicated constructs were incubated with ubiquitin (Ub)-conjugated agarose or agarose beads only (negative control). Precipitates were analyzed by western blotting with α-ING1 or α-ING1 plus α-p53 antibodies. *Lower panel*: western blot confirming ING1 and p53 protein expression. *Control (C)-lanes*: precipitates from untransfected cells. *GFP*: green fluorescence protein.
(TIF)

Figure S8 Sequence alignment of the conserved (common) region of ING1b with a group of four representative PHD proteins and four proteins that contain a ubiquitin-binding-domain (Clustal W program (18)). *Abbreviations: PIP*: PCNA-interacting-protein-domain; *LID*: Lamin-interaction-domain; *PHD*: plant-homeodomain; *UBD*: ubiquitin-binding-domain; *UIM*: ubiquitin-interacting-motif; *NLS*: nuclear localization-sequence; *NTS*: nucleolar targeting-sequence. *Bar height*: degree of conservation of the C_4_HC_3_-residues of the PHD and EL/M ALSE-residues of the UIM.
(TIF)

Figure S9 Lysates of H1299 cells co-transfected with wild-type (wt) or the K_6_R-mutant of p53 and either vector (V), wild-type *ING1b* (wt) or ING1b with a deletion at the ubiquitin-interacting-motif (UIM) of its plant homeodomain (PHD) (*ING1b* ^del(PHD-UIM)^) were precipitated with α-p53 and α-ING1 monoclonal antibodies crosslinked to Protein-G-Sepharose. Precipitates were analyzed by blotting using a mixture of α-p53 and α-ING1. *Lower panels*: western blots with 0.5% of the lysates used in the upper panels to determine expression levels of p53 and protein loading (actin).
(TIF)
