# Expression of Concern: The p53 tumor suppressor is stabilized by inhibitor of growth 1 (ING1) by blocking polyubiquitination

**DOI:** 10.1371/journal.pone.0346639

**Published:** 2026-04-07

**Authors:** The PLOS One Editors

After this article [1] was published, concerns were raised regarding Figs 1 and S7. Specifically,

- In Fig 1C, there appear to be vertical discontinuities between the ING1a and + LC lanes of the Cyclin D1 and Ub panels.
- In Fig 1D, the ING1b + P53 and ING1b lanes of the Cyclin D1 panel appear to be discontinuous with the adjacent background.
- In Fig S7, the GFP lane in the upper-right panel appears similar to the p53 lane of the middle-left panel.

Author KR agreed with the concerns noted in Fig 1C and S7 but did not agree with the concern noted in Fig 1D. They indicated that the + LC lane of Fig 1C represents a control lane added to demonstrate total ubiquitin and cyclin D1 levels increase, confirming proteosomal inhibition by LC treatment. The author KR also stated that this lane does not affect the interpretation of the data.

Author KR also stated that the original, uncropped autoradiograms underlying the figures in this article are no longer available due to the time elapsed since the submission of the article. In the absence of these underlying image data, the above concerns cannot be fully resolved.

The *PLOS One* Editors issue this Expression of Concern to notify readers of the above concerns.
