# Supplementary figures and images for: The p53 Tumor Suppressor Is Stabilized by Inhibitor of Growth 1 (ING1) by Blocking Polyubiquitination
Source: PLoS One. 2011 Jun 22;6(6):e21065. doi: 10.1371/journal.pone.0021065 (PMC3120833; doi:10.1371/journal.pone.0021065)

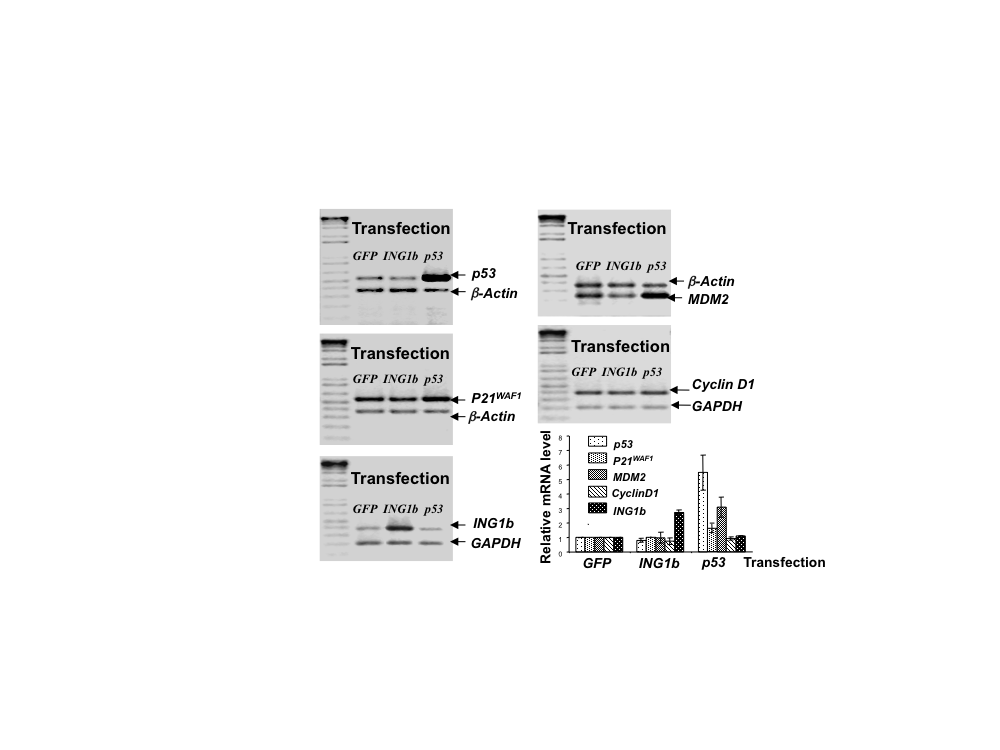

Supplement: Figure S1 — RT-PCR using GAPDH or actin as internal amplification controls was performed to estimate TP53-, P21 WAF1-, ING1b-, MDM2-, or Cyclin D1-mRNA-levels in Hs68 fibroblasts transfected with the indicated constructs. Bar-graph: mean-mRNA-levels of three independent experiments, setting green fluorescence (GFP; negative control) to zero. Error bars: standard deviations. (TIF) [file pone.0021065.s001.tif]

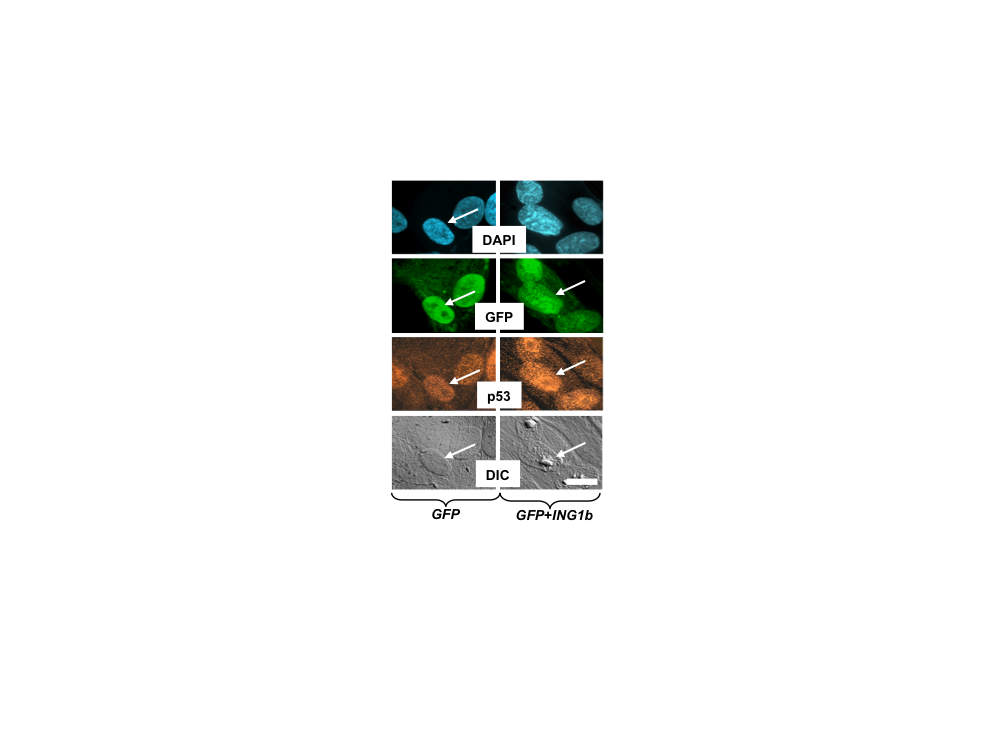

Supplement: Figure S2 — Hs68 fibroblasts injected with the indicated constructs were stained for DNA (DAPI) and p53. Green fluorescence (GFP) identifies injected cells. Arrows: nucleolar morphology in the absence and presence of elevated ING1b. p53 levels were elevated in 55 of 60 cells examined and localization was nuclear, perinuclear and cytoplasmatic. Bar = 10 µm. (TIF) [file pone.0021065.s002.tif]

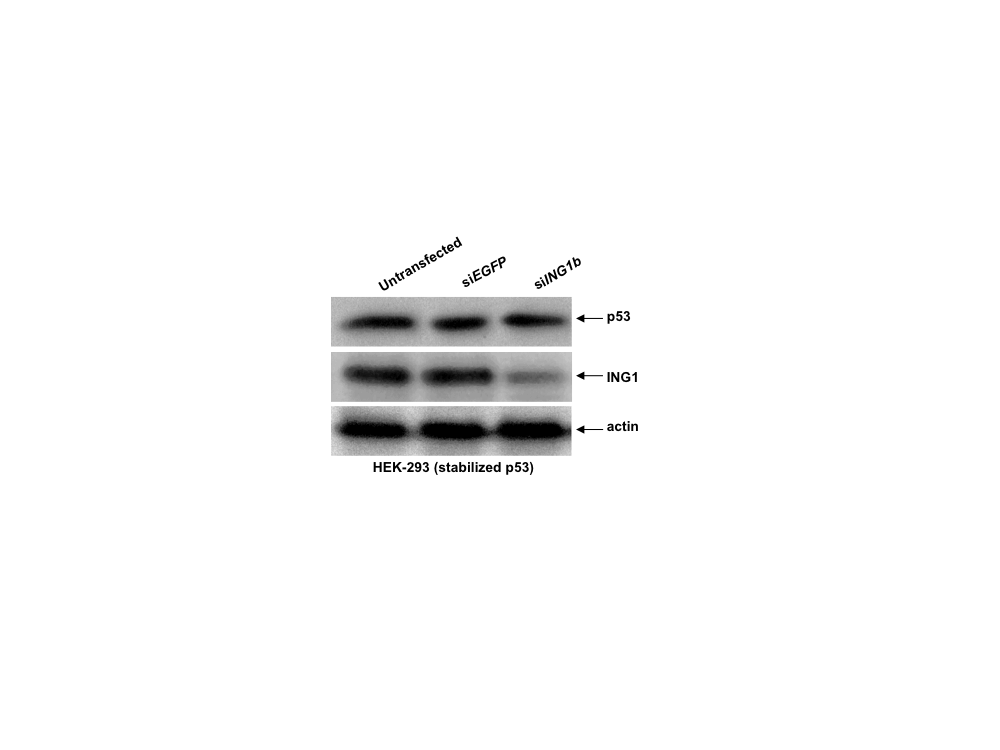

Supplement: Figure S3 — HEK293 cells transfected with the indicated siRNAs were analyzed for p53, ING1b and actin levels. (TIF) [file pone.0021065.s003.tif]

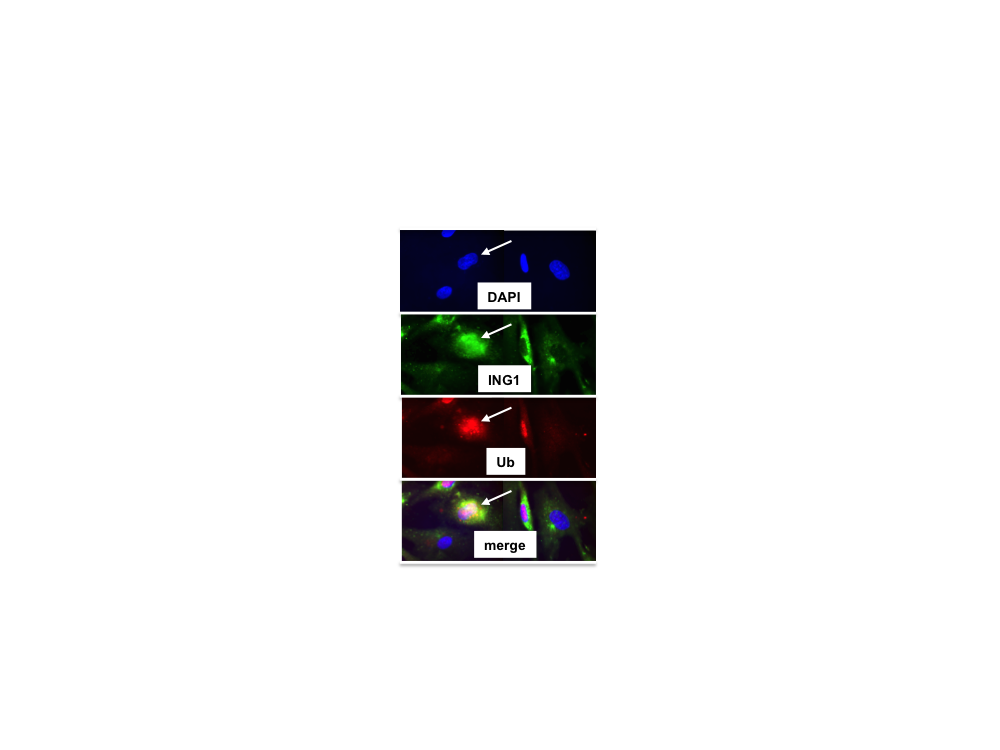

Supplement: Figure S4 — ING1b-transfected Hs68 cells were stained for DNA (DAPI), ING1 and ubiquitin (Ub). The arrow highlights a transfected cell. (TIF) [file pone.0021065.s004.tif]

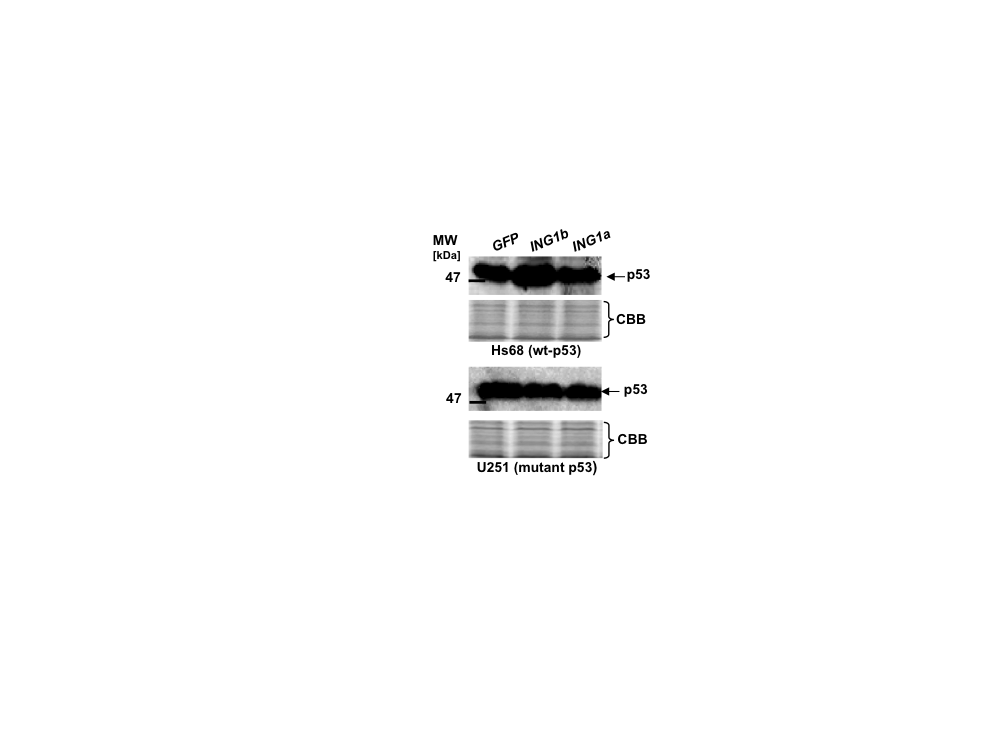

Supplement: Figure S5 — Lysates from cells with wild-type (wt; Hs68) or mutant (U251; containing the p53R273H mutation) p53 infected with the indicated constructs were blotted for p53. CBB-lanes: Coomassie-stained loading controls. Green fluorescence (GFP) indicated infection efficiencies of >95% in all cases. (TIF) [file pone.0021065.s005.tif]

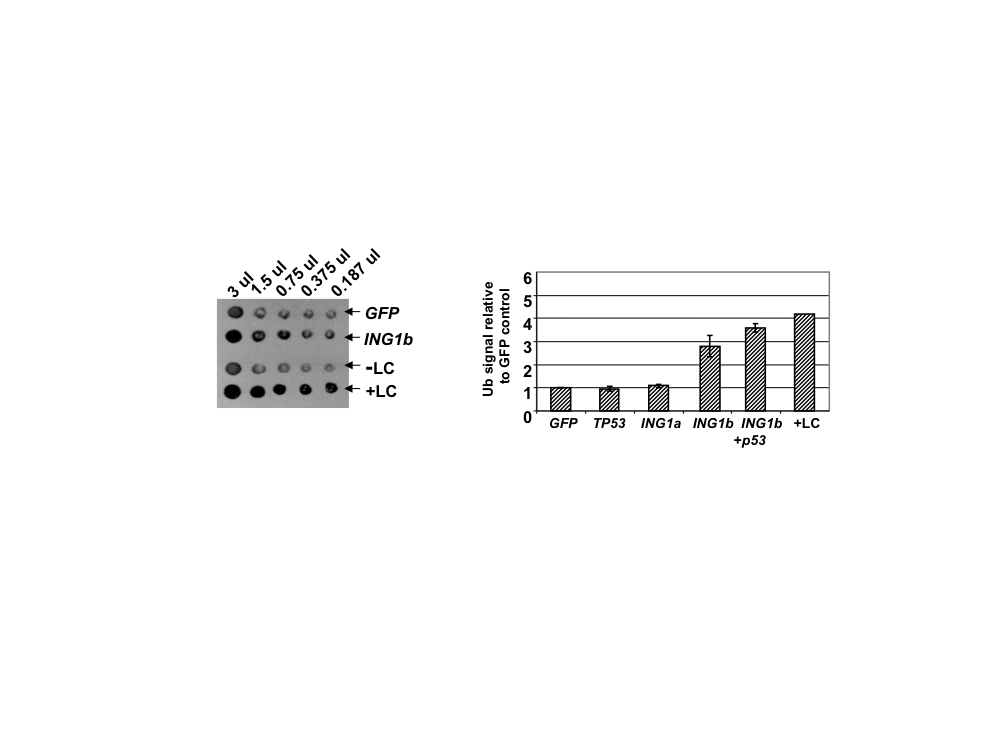

Supplement: Figure S6 — Dot-blot dilution series of lysates from Hs68 fibroblasts treated for 12h with lactacystin (+LC) or left untreated (-LC) served as positive and negative controls, respectively, for lysates from cells infected with the adenoviral constructs indicated. Blots were probed with ubiquitin (Ub) antibodies. Signals from three independent ELISA experiments using Hs68 fibroblasts were quantitated by scanning densitometry and plotted. Error bars: standard deviations. (TIF) [file pone.0021065.s006.tif]

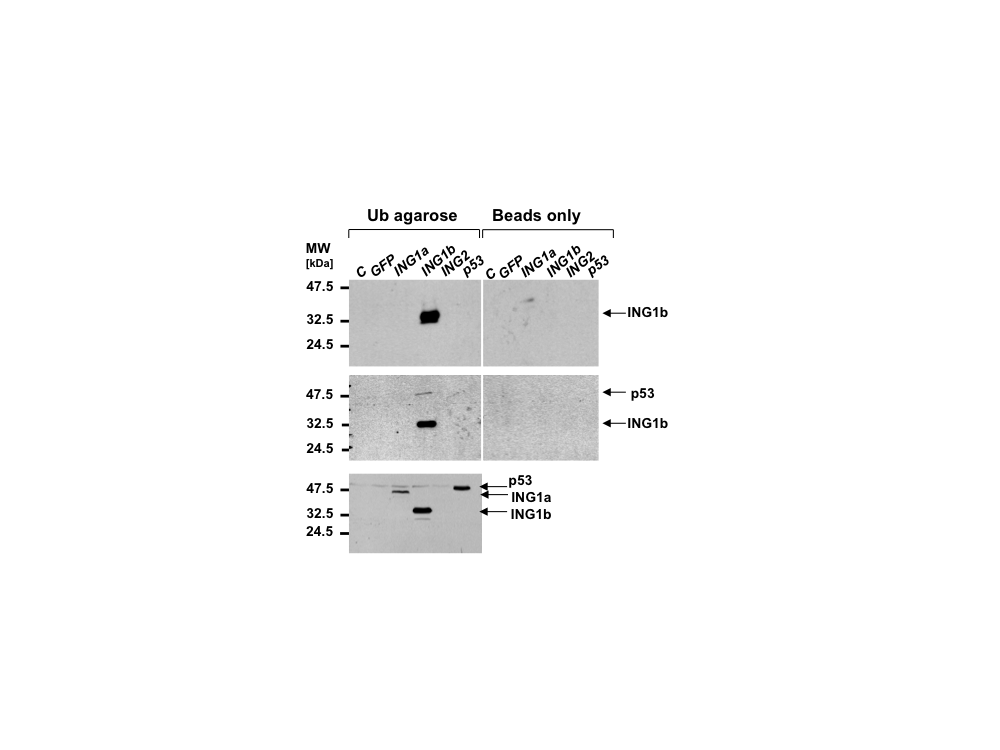

Supplement: Figure S7 — Lysates of HCT116 cells (TP53 -|-) infected with the indicated constructs were incubated with ubiquitin (Ub)-conjugated agarose or agarose beads only (negative control). Precipitates were analyzed by western blotting with α-ING1 or α-ING1 plus α-p53 antibodies. Lower panel: western blot confirming ING1 and p53 protein expression. Control (C)-lanes: precipitates from untransfected cells. GFP: green fluorescence protein. (TIF) [file pone.0021065.s007.tif]

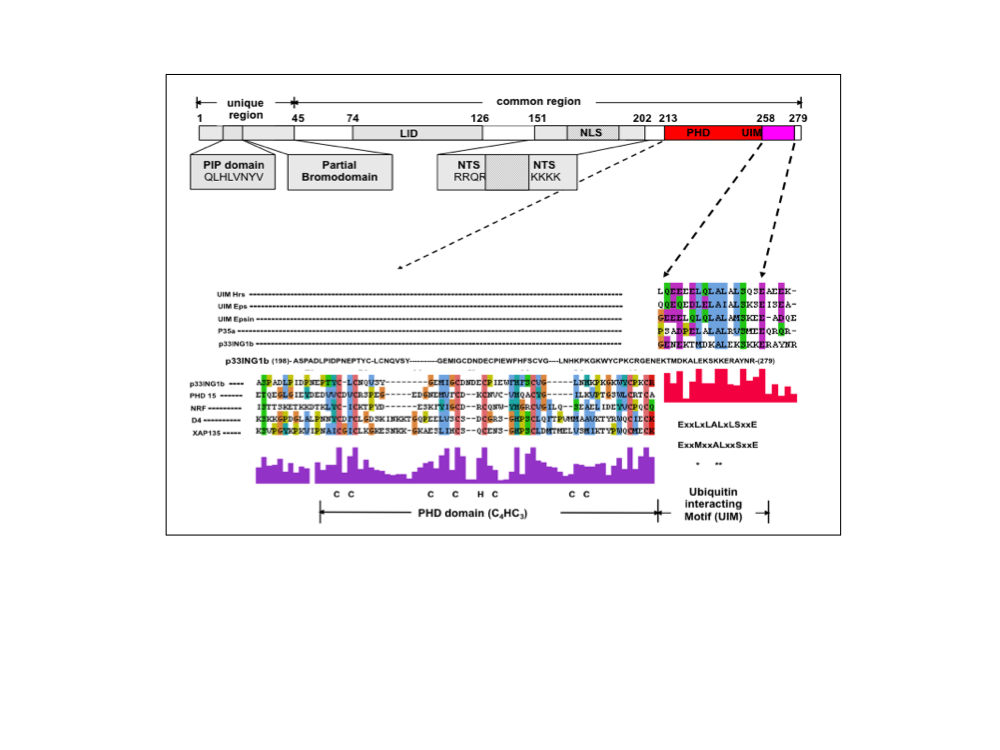

Supplement: Figure S8 — Sequence alignment of the conserved (common) region of ING1b with a group of four representative PHD proteins and four proteins that contain a ubiquitin-binding-domain (Clustal W program (18)). Abbreviations: PIP: PCNA-interacting-protein-domain; LID: Lamin-interaction-domain; PHD: plant-homeodomain; UBD: ubiquitin-binding-domain; UIM: ubiquitin-interacting-motif; NLS: nuclear localization-sequence; NTS: nucleolar targeting-sequence. Bar height: degree of conservation of the C4HC3-residues of the PHD and EL/M ALSE-residues of the UIM. (TIF) [file pone.0021065.s008.tif]

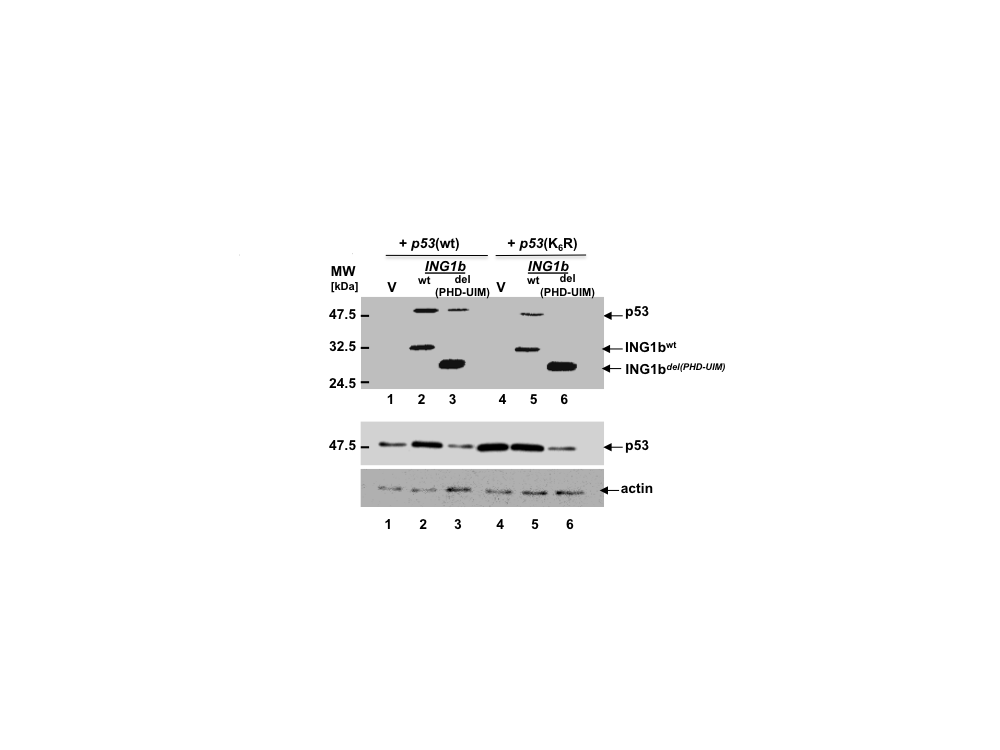

Supplement: Figure S9 — Lysates of H1299 cells co-transfected with wild-type (wt) or the K6R-mutant of p53 and either vector (V), wild-type ING1b (wt) or ING1b with a deletion at the ubiquitin-interacting-motif (UIM) of its plant homeodomain (PHD) (ING1b del(PHD-UIM)) were precipitated with α-p53 and α-ING1 monoclonal antibodies crosslinked to Protein-G-Sepharose. Precipitates were analyzed by blotting using a mixture of α-p53 and α-ING1. Lower panels: western blots with 0.5% of the lysates used in the upper panels to determine expression levels of p53 and protein loading (actin). (TIF) [file pone.0021065.s009.tif]
